# Efficacy and safety analysis of angiotensin receptor neprilysin inhibition(ARNI)in patients with heart failure: a real-world retrospective study

**DOI:** 10.1186/s12872-023-03374-w

**Published:** 2023-07-10

**Authors:** Xiaobo Wang, Jun Pu, Guixia Wang, Hui Xu, Liming Liu, Zhen Li, Ruijie Qin, Xuemei Zhao, Ming Li, Zedong Hao, Houxiang Hu

**Affiliations:** 1 Department of Cardiology, Affiliated Hospital of North Sichuan Medical College, Nanchong, 637000 China; 2 Research Center for High Altitude Medicine of Qinghai University, Xining, 810001 China; 3 Infectious Department, The Third People’s Hospital of Chengdu, Chengdu, 610031 China; 4 Department of Gerontology, Affiliated Hospital of North Sichuan Medical College, Nanchong, China; 5 Undergraduate in clinical medicine, North Sichuan Medical College, Nanchong, China; 6 Shanghai Synyi Medical Technology Co., Ltd, Shanghai, China

**Keywords:** Standard treatment, Angiotensin receptor neprilysin inhibition, Heart failure, Real-world retrospective study

## Abstract

**Background:**

In a large randomized controlled trial (PARADIGM-HF), ARNI has been shown to significantly reduce cardiovascular mortality and hospitalization for patients with reduced ejection fraction in heart failure. This study analyzed the efficacy and safety of ARNI on the basis of various types of heart failure patients in southwestern Sichuan Province.

**Methods:**

This study included patients with heart failure who were treated at the Affiliated Hospital of North Sichuan Medical College from July 2017 to June 2021. This study analyzed the efficacy and safety of ARNI in the treatment of heart failure, and analyzed the risk factors for readmission after ARNI treatment.

**Results:**

After propensity score matching, a total of 778 patients were included in the study. The readmission rate for heart failure in patients treated with ARNI (8.7%) was significantly lower than that in the standard treatment group (14.5%) (P = 0.023). Both the proportion of patients with increased LVEF and with decreased LVEF were higher in the ARNI treatment group than in the conventional therapy group. Compared with receiving standard medical treatment, combined ARNI treatment resulted in a greater reduction in SBP (-10.00, 95%CI: -24.00-1.50 vs. -7.00, 95%CI: -20.00-4.14; P = 0.016) in HF patients. Combination ARNI therapy did not increase the risk of adverse events. The study found that age (> 65 vs. ≤65 years) (OR = 4.038, 95%CI: 1.360-13.641, P = 0.013) and HFrEF (OR = 3.162, 95%CI: 1.028–9.724, P = 0.045) were independent predictors of readmission in HF patients treated with ARNI.

**Conclusion:**

Patients with heart failure treated with ARNI can improve clinical symptoms and reduce the risk of readmitted hospital admission. Age > ~ 65 years and HFrEF were independent predictors of readmission in HF patients treated in ARNI group.

## Introduction

Heart failure (HF) is a major public health problem that imposes an enormous social and economic burden on the world, and about 64.3 million people in the world suffer from HF [1]. In developed countries, the prevalence of diagnosed HF is approximately 1–2% of the general adult population [2]. More than 4 million people in China are affected by HF, with approximately 500 000 new cases diagnosed each year [3]. In addition, the risk of readmission and death in HF patients remains high [4, 5]. Pharmacotherapy is the main treatment option for the treatment of heart failure. Current treatments target hemodynamic changes and neurohumoral composition to slow disease progression and improve symptoms and outcomes. The pathophysiology of HF is associated with persistent activation of the renin-angiotensin-aldosterone system (RAAS), and drugs that inhibit key components of the RAAS are therefore introduced into the clinical pharmacotherapy of HF [6]. The choice of drug therapy in patients with heart failure is based on HF with reduced ejection fraction (HFrEF) and HF with preserved ejection fraction (HFpEF). Angiotensin Converting Enzyme Inhibitor (ACEI), Angiotensin Receptor Blocker (ARB), Mineralocorticoid Receptor Antagonist (MRA), Beta Blocker, Angiotensin Receptor/Enkephalin Enzyme inhibitors (ARNIs) are recommended for the treatment of HFrEF [7, 8]. To date, no treatment has convincingly reduced mortality and morbidity in patients with HFpEF. In recent years, angiotensin receptor-neprilysin inihibitor (ARNI) is more and more widely used in the clinical treatment of heart failure. Compared with ACEI, ARNI reduces HF hospitalization or cardiovascular mortality in patients with HFrEF [9]. A single-center retrospective analysis in Thailand suggested that ARNI use was associated with significantly lower clinical outcomes and symptoms in patients with HFrEF [10].

In China, there is currently a lack of real-world studies analyzing drug therapy in patients with heart failure. To explore the efficacy and safety of ARNI in heart failure patients in Southwest Sichuan Province, we conducted a real-world study based on medical data from the Affiliated Hospital of North Sichuan Medical College in Sichuan Province, China.

## Methods

### Patients

**Fig. 1:**
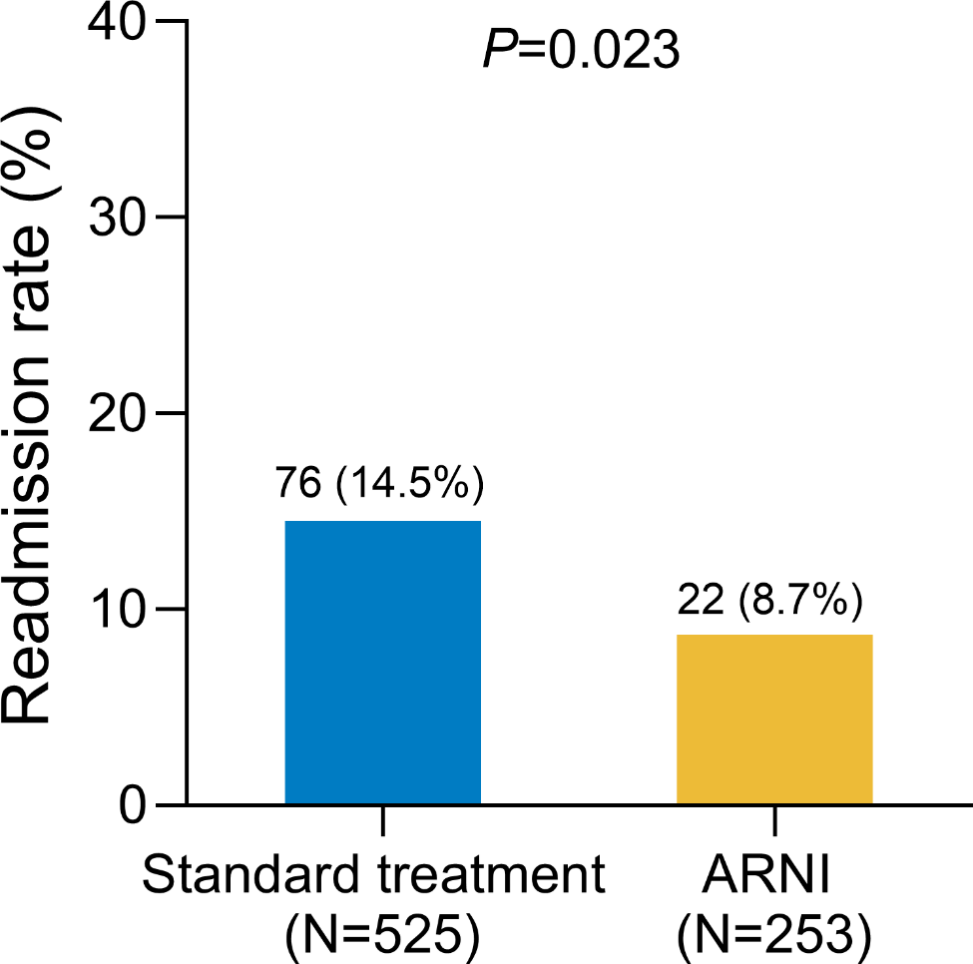
Flow chart of the sduty

This study is a real-world study involving patients with heart failure who were treated at the Affiliated Hospital of North Sichuan Medical College from July 2017 to June 2021. Inclusion criteria include meeting the diagnosis and treatment standards for HF in the “Chinese Heart Failure Diagnosis and Treatment Guidelines 2018”; receiving heart failure drug treatment, including S-V (ARNI), ARB, ACEI, etc.; follow-up for at least 6 months; patients ≥ 18 years old. The study excluded patients with contraindications to the use of HF medications, patients with follow-up less than half a year and patients with missing follow-up data. All patients were divided into standard treatment group and ARNI group according to whether they received combined ARNI treatment or not (Fig. 1). The study compared the efficacy and safety of standard therapy with ARNI therapy and analyzed the risk factors of readmissions in patients with heart failure receiving ARNI therapy. The study was approved by the Ethics Committee of the Affiliated Hospital of North Sichuan Medical College (2022ER291-1), and since the study only involved retrospective analysis of previous clinical data, the requirement for informed consent was waived the Ethics Committee of the Affiliated Hospital of North Sichuan Medical College.

### Variables extraction

The data used in this study were extracted from a database constructed by combining information from multiple data sources, including the Hospital Information System, Laboratory Information Management System, Picture archiving and communication systems, and Electronic Medical Record of the Affiliated Hospital of North Sichuan Medical College. Variables included in the study included patients’ sociodemographic information, drinking history, smoking history, previous medical history, comorbidities, HF-related characteristics, New York Heart Association (NYHA) functional class, laboratory parameters, medication, and other treatments.

### Outcomes and definition

The outcomes of interest in this study were readmission rate, hypotension, and renal impairment. The definition of heart failure refers to the description of heart failure in “Chinese guidelines for the diagnosis and treatment of heart failure 2018“ [11]. Standard treatment was defined as patients receiving β-blockers (Metoprolol succinate sustained-release tablets: 47.5 ~ 95 mg, qd; or Bisoprolol fumarate: 5 ~ 10 mg, qd; or Metoprolol Tartaric acid: 25 ~ 50 mg, bid) + MRA (spironolactone, 20 mg, qd) + angiotensin converting-enzyme inhibitor (ACEI) (Perindopril: 4 ~ 8 mg, qd; or enalapril: 10 ~ 20 mg, qd; or Bellapril: 5 ~ 10 mg, qd) or angiotensin receptor blocker (ARB) (Valsartan: 40 ~ 80 mg, qd; or losartan potassium: 50 ~ 100 mg, qd; or Irbesartan: 0.15 ~ 0.3 g, qd; or Candesartan: 4 ~ 8 mg, qd). The ARNI group was defined as patients treated with β-blockers (Metoprolol succinate sustained-release tablets: 47.5 ~ 95 mg, qd; or Bisoprolol fumarate: 5 ~ 10 mg, qd; or Metoprolol Tartaric acid: 25 ~ 50 mg, bid) + MRA (spironolactone, 20 mg, qd) + ARNI (the initial dose is 25 mg, gradually titrated to 200 mg, bid).

### Statistical analysis

Propensity score matching (PSM) was used to balance measurable confounders between standard treatment group and ARNI group. The propensity score was calculated by logistic regression model with the following covariates age, gender, height, weight, smoking, drinking, medical history, type of HF and NYHA classification. The matching was performed using a 1:2 nearest-neighbor matching protocol with caliper width 0.1. After PSM, statistical analyses were performed using SPSS 26.0 (SPSS Inc., Chicago, IL, USA). Figures were plotted using GraphPad Prism 8.02 (GraphPad Software Inc., San Diego, CA, USA). Continuous variables were described as median and interquartile. Categorical variables were described as number and percentage. Comparisons between standard treatment group and ARNI group were conducted by Wilcoxon rank sum test or Chi-square test. Factors association with readmission rate in ARNI group were screened using univariate logistic regression model, and variables with P value < 0.1 in univariate logistic regression model were further analyzed using multivariate logistic regression model. All tests were two-tail, P value < 0.05 were considered statistically significant.

## Results

### Baseline characteristics of patients with heart failure

Before propensity score matching, a total of 2096 patients with heart failure met the criteria. Among them, 1826 patients received standard therapy and 270 patients received ARNI therapy. There were significant differences in age, sex, height, weight, smoking, drinking, PAD, type of HF, and NYHA classification between the standard care and ARNI treatment groups (Table 1). So, the researchers used propensity score matching to balance the characteristics of the two groups of patients. After propensity score matching, a total of 778 patients were included in the study. There were 525 in the standard treatment group and 253 in the ARNI treatment group. There were no significant differences in patient characteristics between the standard treatment and ARNI-treated groups (Table 1).

**Table 1:** Baseline characteristics

Variables	Unmatched				Matched			
	Total	Standard treatment	ARNI	*P* value	Total	Standard treatment	ARNI	*P* value
Number of patients	2096	1826	270		778	525	253	
Age (years), median (IQR)	71.0 (64.0–77.0)	72.0 (64.0–78.0)	67.0 (56.0-75.3)	**< 0.001**	68.0 (58.0–76.0)	69.0 (60.0–76.0)	68.0 (57.0–76.0)	0.313
Gender, n (%)				**< 0.001**				0.887
Male	1241 (59.2)	1043 (57.1)	198 (73.3)		554 (71.2)	373 (71.0)	181 (71.5)	
Female	855 (40.8)	783 (42.9)	72 (26.7)		224 (28.8)	152 (29.0)	72 (28.5)	
Height (cm), median (IQR)	160.0 (155.0-166.0)	160.0 (155.0-165.0)	163.0 (158.0-169.0)	**< 0.001**	163.0 (158.0-168.0)	163.6 (158.0-168.0)	163.0 (158.0-168.5)	0.854
Weight (kg), median (IQR)	60.0 (53.7–68.0)	60.0 (53.0–67.0)	61.3 (55.0–70.0)	**0.013**	62.2 (55.0–70.0)	63.0 (55.0–70.0)	60.0 (54.5–70.0)	0.167
BMI (kg/m2), median (IQR)	23.4 (21.2–25.4)	23.4 (21.2–25.4)	23.4 (21.0-25.8)	0.791	23.5 (21.3–25.8)	23.7 (21.4–25.9)	23.3 (20.9–25.7)	0.168
Smoking, n (%)	728 (34.7)	594 (32.5)	134 (49.6)	**< 0.001**	352 (45.2)	230 (43.8)	122 (48.2)	0.247
Drinking, n (%)	556 (26.5)	450 (24.6)	106 (39.3)	**< 0.001**	282 (36.2)	185 (35.2)	97 (38.3)	0.399
Medical history, n (%)								
MI	20 (1.0)	16 (0.9)	4 (1.5)	0.314	10 (1.3)	6 (1.1)	4 (1.6)	0.611
Angina	7 (0.3)	7 (0.4)	0 (0.0)	0.605	0 (0.0)	0 (0.0)	0 (0.0)	/
HF	1 (0.0)	1 (0.1)	0 (0.0)	1.000	0 (0.0)	0 (0.0)	0 (0.0)	/
Ischemic stroke	169 (8.1)	152 (8.3)	17 (6.3)	0.253	66 (8.5)	51 (9.7)	15 (5.9)	0.098
Arrhythmia	20 (1.0)	18 (1.0)	2 (0.7)	1.000	5 (0.6)	3 (0.6)	2 (0.8)	0.720
PAD	1471 (70.2)	1248 (68.3)	223 (82.6)	**< 0.001**	605 (77.8)	399 (76.0)	206 (81.4)	0.088
Diabetes	390 (18.6)	342 (18.7)	48 (17.8)	0.708	135 (17.4)	92 (17.5)	43 (17.0)	0.856
Hypertension	928 (44.3)	799 (43.8)	129 (47.8)	0.214	360 (46.3)	242 (46.1)	118 (46.6)	0.886
CKD	38 (1.8)	32 (1.8)	6 (2.2)	0.622	20 (2.6)	14 (2.7)	6 (2.4)	0.807
Renal insufficiency	17 (0.8)	15 (0.8)	2 (0.7)	1.000	8 (1.0)	6 (1.1)	2 (0.8)	1.000
Hyperlipidemia	20 (1.0)	16 (0.9)	4 (1.5)	0.314	7 (0.9)	3 (0.6)	4 (1.6)	0.223
Depression	6 (0.3)	6 (0.3)	0 (0.0)	1.000	0 (0.0)	0 (0.0)	0 (0.0)	/
Type of HF, n (%)				**< 0.001**				0.296
HFrEF	364 (17.4)	245 (13.4)	119 (44.1)		293 (37.7)	190 (36.2)	103 (40.7)	
HFmrEF	315 (15.0)	256 (14.0)	59 (21.9)		172 (22.1)	114 (21.7)	58 (22.9)	
HFpEF	1417 (67.6)	1325 (72.6)	92 (34.1)		313 (40.2)	221 (42.1)	92 (36.4)	
NYHA classification, n (%)				**< 0.001**				0.573
I	323 (15.4)	298 (16.3)	25 (9.3)		82 (10.5)	58 (11.0)	24 (9.5)	
II	866 (41.3)	785 (43.0)	81 (30.0)		252 (32.4)	173 (33.0)	79 (31.2)	
III	714 (34.1)	585 (32.0)	129 (47.8)		338 (43.4)	220 (41.9)	118 (46.6)	
IV	193 (9.2)	158 (8.7)	35 (13.0)		106 (13.6)	74 (14.1)	32 (12.6)	

IQR, interquartile range; BMI, body mass index; MI, myocardial infarction; PAD, peripheral arterial disease; CKD, chronic kidney disease; HF, heart failure; NYHA, New York Heart Association

### Comparison of readmission rates between standard treatment and ARNI treatment groups

**Fig. 2:**
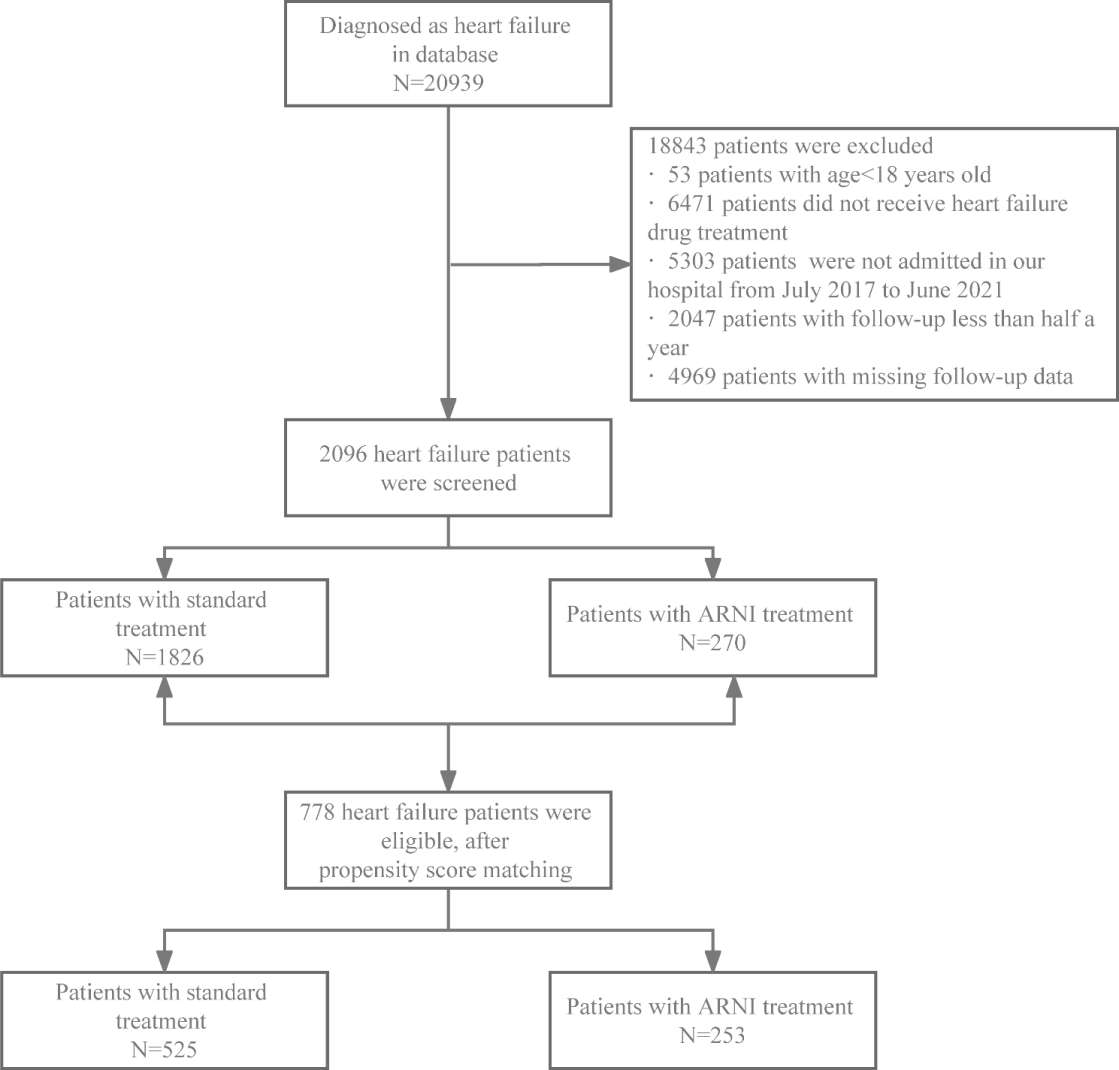
Compare the re-hospitalization rate between two group

Seventy-six patients (14.5%) were readmitted for heart failure in the standard-therapy group and 22 patients (8.7%) in the ARNI-treated group. There was a significant difference in the readmission rate between the two groups (P = 0.023) (Fig. 2).

*Comparison of clinical characteristics of patients in standard treatment group and ARNI treatment group before and after treatment*.

To analyze the effect of standard drug therapy and combined ARNI on HF patients, the changes of biochemical parameters and imaging parameters of the two groups of patients before and after treatment were compared. The results showed that there were significant differences in △LVEF, △LVEDD, △SBP, and △NT-proBNP between the two groups (Table 2). Both the proportion of patients with increased LVEF and the proportion of patients with decreased LVEF were higher in the ARNI treatment group than in the standard treatment group. In the ARNI treatment group, a greater proportion of patients with decreased LVEDD than in the standard treatment group, and a lower proportion of patients with an increased LVEDD than in the standard treatment group. Compared with receiving standard medical treatment, combined ARNI treatment resulted in a greater reduction in SBP (-10.00, 95%CI: -24.00-1.50 vs. -7.00, 95%CI: -20.00-4.14; P = 0.016) in HF patients. Although the ΔNT-proBNP of the two groups was statistically significant (standard treatment group: 0.00, 95%CI: -768.76-0.00; ARNI treatment group: 0.00, 95%CI: -2185.00-0.00; P = 0.010), the median value of ΔNT-proBNP was 0, and the 95% confidence interval range of the ARNI treatment group was larger (Table 2).

**Table 2:** Compare the change of key clinical features between standard treatment group and ARNI group

Items	Standard treatment (N = 525)	ARNI (N = 253)	*P* value
△NYHA, No. (%)			0.827
Reduced	9 (1.7)	3 (1.2)	
Unchanged	502 (95.6)	244 (96.4)	
Elevated	14 (2.7)	6 (2.4)	
△LVEF, No. (%)			**0.002**
Reduced	36 (6.9)	35 (13.8)	
Unchanged	387 (73.7)	162 (64.0)	
Elevated	102 (19.4)	56 (22.1)	
△LVEDD, No. (%)			**0.025**
Reduced	55 (10.5)	44 (17.4)	
Unchanged	401 (76.4)	177 (70.0)	
Elevated	69 (13.1)	32 (12.6)	
△DBP, median (IQR)	-5.00 (-14.50-3.00)	-6.00 (-17.00-2.00)	0.228
△SBP, median (IQR)	-7.00 (-20.00-4.14)	-10.00 (-24.00-1.50)	**0.016**
△Serum creatinine, median (IQR)	0.00 (0.00–0.00)	0.00 (0.00–0.00)	0.275
△eGFR, median (IQR)	0.00 (0.00-0.85)	0.00 (0.00–0.00)	0.600
△NT-proBNP, median (IQR)	0.00 (-768.76-0.00)	0.00 (-2185.00-0.00)	**0.010**
△serum potassium, median (IQR)	0.00 (-0.17-0.50)	0.09 (-0.05-0.52)	0.089

△post treatment level - baseline level. NYHA, New York Heart Association; LVEF, left ventricular ejection fraction; LVEDD, left ventricular end-diastolic diameter; DBP, diastolic blood pressure; IQR, interquartile range; SBP, systolic blood pressure; eGFR, estimated glomerular filtration rate; NT-proBNP, pro-brain natriuretic peptide

### Compare adverse events between standard treatment group and ARNI treatment group

There were no significant differences in the overall incidence of adverse events (1.5%vs. 2.0%, P = 0.766) and the incidence of hypotension (1.3%vs. 1.6%, P = 0.755) and renal injury (0.2%vs. 0.4%, P = 0.545) between the standard-care and ARNI-treated patients (Table 3).

**Table 3:** Compare adverse events between two group

Adverse events	Standard treatment (N = 525)	ARNI (N = 253)	*P* value
Hypotension	7 (1.3)	4 (1.6)	0.755
Renal impairment	1 (0.2)	1 (0.4)	0.545
Total	8 (1.5)	5 (2.0)	0.766

### Logistics regression analysis of risk factors for readmission in ARNI-treated patients

Univariate logistic regression analysis found that age (> 65 vs. ≤65 years), HFrEF, NYHA classification, serum creatinine (abnormal vs. normal), and eGFR (abnormal vs. normal) were all associated with readmissions in ARNI-treated patients (all P < 0.1) (Table 4). The study included all variables with P < 0.1 into multivariate logistic regression analysis and found that age (> 65 vs. ≤65 years) (OR = 4.038, 95%CI: 1.360-13.641, P = 0.013) and HFrEF (OR = 3.162, 95%CI: 1.028–9.724, P = 0.045) were independent predictors of readmission in HF patients treated with ARNI (Tables 4 and 5).

**Table 4:** Factors association with readmission in ARNI group

Items	Univariate logistic regression model			
	*P* value	OR	95%CI	
			Lower	Higher
Age (> 65 vs. ≤65 years)	**0.018**	3.816	1.253	11.624
Sex (female vs. male)	0.120	0.371	0.106	1.294
BMI (> 24 vs. ≤24 kg/m^2^)	0.784	1.131	0.470	2.723
Smoking	0.136	1.993	0.805	4.933
Drinking	0.243	1.686	0.701	4.054
MI	0.274	3.619	0.360	36.346
Ischemic stroke	0.999	-	-	-
Arrhythmia	0.999	-	-	-
PAD	0.960	1.029	0.332	3.196
Diabetes	0.662	0.754	0.213	2.670
Hypertension	0.151	0.505	0.198	1.283
CKD	0.999	-	-	-
Renal insufficiency	0.999	-	-	-
Hyperlipidemia	0.999	-	-	-
Type of HF				
HFpEF	1 (ref)			
HFrEF	**0.092**	2.513	0.860	7.347
HFmrEF	0.714	1.289	0.331	5.011
NYHA classification	**0.027**	1.934	1.076	3.476
LVEDD (abnormal vs. normal)	0.264	1.801	0.641	5.062
DBP (abnormal vs. normal)	0.617	1.262	0.507	3.142
SBP (abnormal vs. normal)	0.196	1.806	0.736	4.432
Serum creatinine (abnormal vs. normal)	**0.040**	2.544	1.044	6.200
eGFR (abnormal vs. normal)	**0.014**	3.053	1.257	7.416
NT-proBNP (abnormal vs. normal)	0.708	1.220	0.432	3.449
Serum potassium (abnormal vs. normal)	0.677	0.802	0.284	2.265

OR, odds rate; CI, confidence interval; BMI, body mass index; MI, myocardial infarction; PAD, peripheral arterial disease; CKD, chronic kidney disease; HF, heart failure; NYHA, New York Heart Association; LVEDD, left ventricular end-diastolic diameter; DBP, diastolic blood pressure; SBP, systolic blood pressure; eGFR, estimated glomerular filtration rate; NT-proBNP, pro-brain natriuretic peptide

**Table 5:** Factors independently associated with readmission in ARNI group

Items	Multivariate logistic regression model			
	*P* value	OR	95%CI	
			Lower	Higher
Age (> 65 vs. ≤65 years)	**0.013**	4.308	1.360	13.641
Type of HF				
HFpEF	1 (ref)			
HFrEF	**0.045**	3.162	1.028	9.724
HFmrEF	0.702	1.319	0.319	5.454
Serum creatinine (abnormal vs. normal)	0.664	0.602	0.061	5.969
eGFR (abnormal vs. normal)	0.173	4.824	0.502	46.341

OR, odds rate; CI, confidence interval; eGFR, estimated glomerular filtration rate

## Discussion

HF has become a global health problem due to the need for frequent hospital admissions, inability to work during periods of decompensation, high cost of care with both pharmacological and non-pharmacological treatments, and high mortality. How to improve the long-term survival and functional ability of patients with heart failure is one of the focal issues to be solved in clinical practice. Real-world assessments of the treatment effects of different drug regimens can provide useful information and enhance our understanding of the benefits-risks of treatment regimens in real clinical practice. This study compared the efficacy and safety of conventional drug therapy with ARNI therapy in real-world clinical practice in patients with heart failure in Southwest Sichuan province. The results found that combined ARNI can more effectively reduce SBP, LEVF, NT-proBNP and LVEDD in patients with heart failure, and can significantly reduce the risk of readmission, without increasing the risk of adverse events.

Based on the results of large randomized controlled trials, ARNI is recommended for initial treatment of HFrEF patients or after switching HFrEF patients from a conventional RAS antagonist [12], and its therapeutic effect has been confirmed by some clinical trials. A sub-analysis of the PARADIGM-HF trial results confirmed the benefit of ARNI initiation in patients with HFrEF, LVEF ≤ 35%, and NYHA II-IV symptoms [13]. Results of a double-blind, randomized, controlled trial showed that ARNI was superior to ACEI in reducing the risk of death and hospitalization for HF in patients with HFrEF. The researchers demonstrated a 20% reduction in the composite risk of cardiovascular death or hospitalization for heart failure and a 16% reduction in the relative risk of all-cause mortality in patients with HFrEF treated with ARNI. Furthermore, the patients had a significant 3 mmHg reduction in blood pressure and a higher quality of life score at 8 months of ARNI treatment [9]. ARNI was safe and well tolerated in patients admitted for acute decompensated HF with or without a history of HF, ACEI or ARB therapy. Compared with enalapril, ARNI can significantly reduce NT-proBNP and improve clinical outcomes [14]. A phase 2 double-blind randomised controlled trial found that compared with valsartan, NT-proBNP was significantly reduced at 12 weeks after ARNI in patients with HFpEF. The findings also suggest that HFpEF patients taking ARNI had significantly greater blood pressure reductions than those taking valsartan at 12- and 36-week intervals. ARNI is well tolerated, with adverse effects similar to valsartan [15]. However, in patients with HFpEF, ARNI did not significantly reduce total hospitalizations for heart failure and mortality from cardiovascular causes [16]. In our study, we also found an interesting result that the proportion of patients with increased LVEF and decreased LVEF in the ARNI treatment group were higher than those in the standard treatment group. The potential reason for this result may be due to the low compliance of patients with medical orders. Patients receiving ARNI treatment often have lower blood pressure, and these patients often start with lower doses of ARNI and do not follow medical orders to receive target doses of ARNI, which may lead to a decrease in LVEF. In another study, we analyzed the treatment adherence of heart failure patients to the guided determined measurement therapy (GDMT). The study found that patients with heart failure have lower adherence to GDMT treatment. The manuscript has been received by Reviews in Cardiovascular Medicine.

ARNI can effectively improve the symptoms of HF patients and reduce the risk of readmission in our study, and we also found that the safety of ARNI is trustworthy. There were no significant differences in the incidence of hypotension and renal injury between the standard-care and ARNI-treated patients. The safety of ARNI has been confirmed by several studies. A systematic review and meta-analysis found that compared with the renin–angiotensin–aldosterone system inhibitor, patients treated with ARNI had lower incidence of composite renal impairment, ESRD, drug discontinuation due to renal events, severe hyperkalemia and a slower eGFR decline [17]. A randomized, double-blind trial compared the renal effects of ARNI or valsartan in patients with HFpEF. The researchers found that compared with valsartan, ARNI reduced the risk of adverse renal events in patients and slowed the decline in eGFR [18]. In HFrEF patients, ARNI delayed eGFR decline and improved cardiovascular outcomes compared with enalapril [19]. The risk of hypotension is one of the main reasons for ARNI intolerance in HF patients. A single-center retrospective study suggested that the primary reason for ARNI resistance in HFrEF patients was hypotension [20]. A multicenter, randomized, double-blind, active-controlled trial included HFrEF patients hospitalized for acute decompensated heart failure at 129 sites in the United States. The study compared the efficacy and safety of in-hospital initiation of ARNI and enalapril. The findings showed that there were no significant differences in the incidence of symptomatic hypotension, worsening renal function, hyperkalemia, and angioedema between the two groups [21]. The PARADIGM-HF trial demonstrated that hypotension was more common in heart failure patients treated with ARNI compared with enalapril. The study also found that individuals with hypotensive events were older, had lower blood pressure at randomization, and were more likely to use an implantable cardioverter-defibrillator [22].

This study analyzed the risk factors for readmission in HF patients treated with ARNI and found that age (> 65 vs. ≤65 years) and HFrEF were independent predictors of readmission in HF patients treated with ARNI. In HFrEF, predictors of readmission or death included older age, increased heart rate, lower systolic blood pressure, history of CKD, history of diabetes, coronary artery disease, and smoking [23]. The study found that older age was an independent predictor of 90-day readmission in HF patients [24]. HFrEF is associated with high hospitalization and mortality [25]. A clinical study based on Japanese heart failure patients showed that HFrEF was a risk factor for readmission in heart failure patients [26]. Based on our findings, clinicians can pay more attention to patients aged > 65 years with a diagnosis of HFrEF, such as increasing the frequency of follow-up and the range of monitoring indicators, which may improve clinical outcomes and reduce the risk of readmission.

This study has some limitations. First, this study was a single-center, retrospective study with a small sample size. Second, the dose of ARNI was not analyzed by group. Third, the follow-up information of some patients was obtained through telephone follow-up, and there may be information bias. Therefore, more randomized controlled trials or prospective studies are needed to further verify the efficacy and safety of ARNI.

In conclusion, compared with standard treatment, HF patients in ARNI treatment group can improve the clinical symptoms of patients and reduce the risk of readmission, and do not increase the risk of adverse events. Age > 65 years and HFrEF were independent predictors of readmission in HF patients receiving combined ARNI therapy.

## Data Availability

The data that support the findings of this study are available from Affiliated Hospital of North Sichuan Medical College but restrictions apply to the availability of these data, which were used under license for the current study, and so are not publicly available. Data are however available from the corresponding author upon reasonable request and with permission of Affiliated Hospital of North Sichuan Medical College.
